# The Effect of Clinical, Radiological and Surgical Factors on Postoperative Complications in Solitary Extremity Schwannomas

**DOI:** 10.3390/jcm15031235

**Published:** 2026-02-04

**Authors:** Hüseyin Sina Coşkun, Furkan Erdoğan, Bedirhan Albayrak, Abdurrahman Murat Yıldırım, Veli Süha Öztürk, Nevzat Dabak

**Affiliations:** 1 Department of Orthopaedics and Traumatology, Ondokuz Mayıs University, Samsun 55100, Turkey; sina.coskun@omu.edu.tr (H.S.C.); erdogan27@yahoo.com (F.E.); ndabak@gmail.com (N.D.); 2 Department of Orthopaedics and Traumatology, Samsun Training and Research Hospital, Samsun 55090, Turkey; 3 Department of Orthopaedics and Traumatology, Bafra State Hospital, Samsun 55400, Turkey; muratyildirim.1989@hotmail.com; 4 Department of Radiology, Ondokuz Mayıs University, Samsun 55270, Turkey; velisuha.ozturk@gmail.com

**Keywords:** schwannoma, peripheral nerve tumor, extremity, postoperative complication, fascicular involvement, MRI, tru-cut biopsy

## Abstract

**Background/Objectives**: To evaluate the clinical and MRI characteristics of benign solitary schwannomas of the extremities, compare pre- and postoperative neurological symptoms, and identify preoperative and intraoperative risk factors for postoperative complications. **Methods:** A retrospective review was conducted on 47 patients with histopathologically confirmed benign solitary schwannomas of the extremities who underwent surgical excision. Demographic data, MRI characteristics (tumor volume, perilesional edema, and degenerative changes such as cystic components or intratumoral hemorrhage), fascicular relationship, and use of tru-cut biopsy were recorded. Pre- and postoperative neurological symptoms were compared. Univariate logistic regression analysis was performed to identify factors associated with postoperative complications. **Results**: The mean age was 38.6 ± 15 years, and the mean follow-up period was 109.8 ± 65.1 months. Lesions were predominantly located in the upper extremity (65.9%), with a mean volume of 9.6 ± 4.8 cm^3^; perilesional edema and/or degenerative changes were present in 53.1% of cases. Postoperative complications occurred in 19.1% of patients, with intrafascicular involvement being a significant predictor (OR = 5.4, *p* = 0.037) and a positive preoperative Tinel’s sign showing a trend toward significance (OR = 4.2, *p* = 0.084). Tumor volume, perilesional edema, degenerative changes, tru-cut biopsy, and anatomical location were not significantly associated with complications. At final follow-up, pain remission was 82.1%, and paresthesia improvement was 63.6%. **Conclusions**: Intrafascicular involvement was associated with postoperative complications in univariate analysis, whereas preoperative MRI characteristics, biopsy, and Tinel’s sign showed no predictive value for postoperative risk.

## 1. Introduction

Schwannomas are benign, slow-growing, and non-invasive peripheral nerve sheath tumors originating from differentiated Schwann cells. Although more frequently observed in the upper extremities, they can occur in any anatomical region. On magnetic resonance imaging (MRI), they are often accompanied by degenerative changes such as cyst formation, fibrosis, and calcification secondary to hemorrhage [1].

Postoperative neurological complication rates following schwannoma excision have been reported to range from 2% to 80% in the literature [2,3,4,5]. This wide variation suggests that clinical and radiological factors may play a decisive role in postoperative outcomes. Indeed, several studies have reported potential associations between complication risk and variables such as tumor size, fascicular involvement, anatomical location (upper vs. lower extremity), preoperative neurological status, and MRI findings, including degenerative changes or perilesional edema [5,6,7,8].

Another factor that may influence the risk of postoperative complications is the diagnostic process itself. In peripheral nerve sheath tumors, there are differing opinions in the literature regarding whether preoperative tru-cut biopsy, used to distinguish benign from malignant lesions, may have indirect effects on surgical outcomes [9]. Schwannomas are encapsulated, typically extrafascicular benign peripheral nerve sheath tumors that displace rather than infiltrate adjacent nerve fascicles, allowing for fascicle-sparing enucleation in most cases. In contrast, neurofibromas lack a true capsule and infiltrate nerve fascicles and axons with a heterogeneous cellular composition, which makes complete excision more challenging and increases the risk of postoperative neurological deficits [10].

The primary goal of surgical treatment in benign peripheral nerve sheath tumors is complete tumor removal while preserving neural function. Although en bloc nerve resection with subsequent reconstruction was historically considered the standard approach, advances in microsurgical techniques have shifted current practice toward fascicle-sparing tumor excision to minimize iatrogenic nerve injury. Consequently, the intraoperative morphological relationship between the tumor and adjacent healthy nerve fascicles has become the key determinant of surgical strategy and technical complexity, forming the basis of the present study [11].

Although most peripheral schwannomas can be surgically excised without causing major complications, some patients may experience new-onset or persistent neurological symptoms in the postoperative period [2]. Therefore, identifying preoperative risk factors is essential for optimizing surgical planning and managing patient expectations. The present study investigated clinical, radiological, and surgical factors associated with postoperative complications in benign solitary schwannomas of the extremities.

## 2. Materials and Methods

This retrospective, single-center study included patients who underwent surgical treatment for benign solitary schwannomas of the extremities between 1996 and 2023. The study was approved by the local ethics committee of the institution and conducted in accordance with the principles of the Declaration of Helsinki (Ondokuz Mayıs University Institutional Review Board, Approval No: 2025/363).

Demographic characteristics, tumor location and volume, MRI findings, associated nerve segment, history of tru-cut biopsy, pre- and postoperative neurological symptoms, and postoperative complications were systematically recorded. In addition, radiological assessments included a detailed evaluation of tumor morphology, its relationship with surrounding soft tissues, and the presence of degenerative changes or perilesional edema. Neurological findings were categorized according to their clinical relevance to allow a consistent and comparable analysis between the preoperative and postoperative periods, thereby improving the reliability of outcome assessment. Inclusion criteria were: age ≥18 years, histopathologically confirmed diagnosis of benign solitary schwannoma, lesion located in the upper or lower extremity, availability of preoperative MRI, and a minimum postoperative follow-up of 12 months. Exclusion criteria included plexiform schwannomas, malignant peripheral nerve sheath tumors (MPNST), neurofibromatosis type 1 or type 2 (NF1/NF2), schwannomatosis, spinal or paraspinal lesions, and incomplete clinical or imaging data.

An experienced musculoskeletal radiologist reviewed all preoperative MRIs. Tumor volume was calculated using the following formula based on MRI measurements: Volume (cm^3^) = Width × Height × Length × 0.52, where 0.52 is a shape coefficient commonly applied for ellipsoid lesions in radiological measurements.

**Figure 1:**
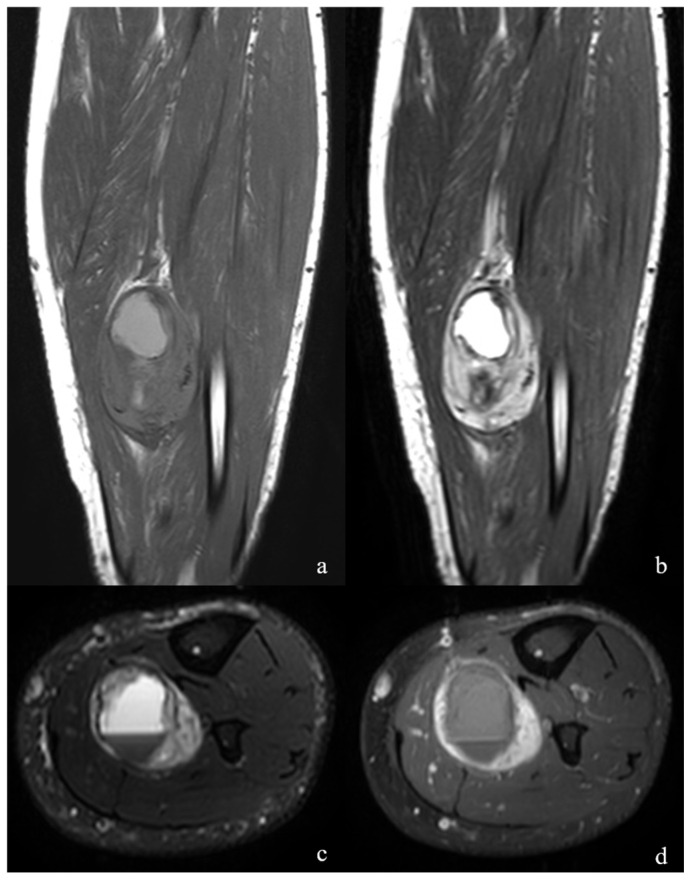
(**a**). The coronal T1-weighted images of a 50-year-old man revealed a well-shaped mass within the muscle planes of the deep posterior compartment at the mid-crural level, exhibiting an isointense signal peripherally and hyperintense hemorrhagic areas centrally. (**b**,**c**). The central hemorrhagic cystic component was clearly delineated on coronal T2-weighted and axial fat-saturated T2-weighted images. (**d**). Postcontrast axial T1-weighted sequences demonstrated marked enhancement in the peripheral solid portion of the lesion.

**Figure 2:**
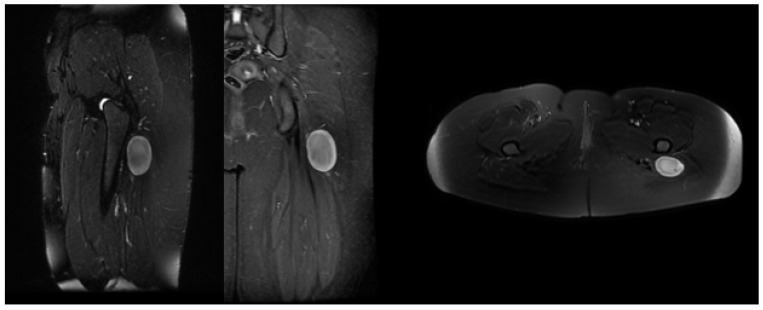
Sagittal, coronal, and axial T2-weighted MRI images of a 29-year-old female patient demonstrating imaging findings consistent with schwannoma. The patient presented with preoperative paresthesia and pain, which regressed following complete surgical excision of the lesion in the postoperative period.

Perilesional edema was defined as a hyperintense signal surrounding the tumor on T2-weighted or STIR sequences, extending into adjacent muscle planes. This definition was adapted from previous MRI studies on peripheral and vestibular schwannomas. Degenerative changes were defined on MRI as heterogeneous signal patterns suggestive of cystic degeneration, hemorrhage, or fibrosis/hyalinization, characterized by focal hyperintense or hypointense areas on T1- and T2-weighted sequences. These imaging findings were considered radiological correlates of histopathological degeneration (e.g., necrosis, fibrosis, or hyalinization). As these findings frequently coexisted, subpatterns were not analyzed separately. At least two of the characteristic MRI features of peripheral nerve sheath tumors—target sign, split fat sign, absence of a lobular shape, and abnormal signal changes along the involved nerve tract—were present in all cases (Figure 1 and Figure 2). Additionally, the specific peripheral nerve segment involved (e.g., median, ulnar, radial, tibial, sciatic, or brachial plexus) was identified for each case.

The decision to perform a tru-cut biopsy was made following multidisciplinary tumor board evaluation in cases presenting with atypical MRI findings or suspected malignancy. All biopsy-proven cases were histopathologically confirmed as benign solitary schwannoma before inclusion. All specimens were histopathologically evaluated by a pathologist experienced in evaluating musculoskeletal and peripheral nerve sheath tumors. The diagnosis of schwannoma was established based on characteristic morphological features, including Antoni A/B areas, the presence of a capsule, and Verocay bodies; in cases requiring differential diagnosis, S-100 and SOX10 positivity, Ki-67 to support a low proliferative index, and additional appropriate immunohistochemical markers were utilized to distinguish schwannoma from neurofibroma and malignant peripheral nerve sheath tumors. Through this comprehensive evaluation, all lesions were reliably confirmed to be schwannomas and not neurofibromas.

### 2.1. Surgical Technique

All surgical procedures were performed by orthopedic surgeons experienced in peripheral nerve surgery. All patients underwent intracapsular resection. When feasible, a pneumatic tourniquet was applied to minimize intraoperative bleeding and to enhance visualization under the operating microscope. Surgical exposure was achieved through an incision centered over the lesion and extended along the anatomical course of the affected nerve, enabling clear identification of the proximal and distal nerve segments. A longitudinal opening of the epineurium was created at a safe distance from intact fascicles, followed by meticulous dissection within the epineurial plane. An en bloc resection of the tumor was attempted whenever feasible. En bloc excision was intentionally avoided in cases where the capsule was firmly adherent to the epineurium. After tourniquet release, careful hemostasis was performed, suction drainage was placed, and the operated extremity was kept elevated during the early postoperative period.

Preoperative and postoperative neurological symptoms (paresthesia, hypoesthesia, pain, motor deficit, Tinel’s sign) were recorded in detail. These were categorized as resolved, persistent, or new-onset during follow-up. Postoperative complications—defined as any new or worsened neurological deficit, wound problem, or local recurrence—were evaluated at the 12-month follow-up. This endpoint was considered the primary outcome variable for analysis. Transient symptoms that resolved within 3 months were not regarded as persistent complications. MRI was performed to assess possible tumor recurrence in cases with new or worsening neurological findings.

### 2.2. Statistical Analysis

Categorical variables were compared using Fisher’s exact or Pearson chi-square tests. The normality of continuous variables was assessed using the Kolmogorov–Smirnov test; variables that did not meet normality assumptions were converted into categorical variables for analysis. Logistic regression analysis was performed to identify independent predictors of postoperative complications. Results are presented as odds ratios (OR) with 95% confidence intervals (CI); *p* < 0.05 was considered statistically significant.

## 3. Results

Forty-seven patients with benign solitary schwannomas of the extremities were included in the study. The mean age was 38.6 years, and the majority of lesions were located in the upper extremity. Demographic, radiological, and surgical characteristics are summarized in Table 1. In terms of anatomical distribution, schwannomas were most frequently located in the median nerve (n = 14) and brachial plexus (n = 3). Other involved nerves included the radial (n = 9), tibial (n = 7), ulnar (n = 3), common peroneal (n = 4), sciatic (n = 2), musculocutaneous (n = 2), saphenous (n = 2), and sural (n = 1) nerves.

Overall, nine patients (19.1%) developed postoperative complications. The detailed distribution is summarized in Table 2.

Overall, postoperative complications were observed in a minority of patients, with sensory disturbances constituting the most common subtype. The majority of these complications were mild in nature and predominantly involved hypoesthesia or paresthesia, whereas motor deficits were relatively uncommon. The distribution of complications across different nerve segments did not demonstrate a statistically significant pattern, suggesting that the occurrence of postoperative deficits was not strongly dependent on the specific anatomical nerve involved. Postoperative neurological improvement was notable: most preoperative sensory complaints improved or resolved, while motor deficits persisted in a few cases. Detailed comparisons of pre- and postoperative symptoms are presented in Table 3.

Among patients with postoperative complications, the most frequently affected nerves were the median nerve (n = 2) and brachial plexus (n = 2), followed by single cases involving the radial, ulnar, tibial, sciatic, and saphenous nerves. However, the nerve-specific distribution was not statistically significant (*p* = 0.386). In univariate logistic regression analysis, intrafascicular location (OR, 5.4; 95% CI, 1.4–22.1; *p* = 0.037) was significantly associated with an increased risk of postoperative complications. Preoperative Tinel’s sign also demonstrated a trend toward significance (OR, 4.2; 95% CI, 0.81–23.9; *p* = 0.084). In contrast, the presence of perilesional edema (OR, 3.5; 95% CI, 0.8–15.6; *p* = 0.091), degenerative changes (OR, 3.1; 95% CI, 0.6–14.4; *p* = 0.140), and tru-cut biopsy (OR, 3.1; 95% CI, 0.7–15.1; *p* = 0.141) were not significantly associated with postoperative complications. Although degenerative changes tended to occur in larger tumors, this association was not statistically significant (*p* = 0.567), and no clear relationship was found between perilesional edema and tumor volume (*p* = 0.091) (Table 4).

## 4. Discussion

Schwannomas are typically slow-growing, benign peripheral nerve tumors that can usually be managed with microsurgical techniques aimed at preserving neurological function. Despite their generally favorable prognosis, postoperative complications may occur, particularly in lesions with close anatomical relationships to functional nerve fascicles. In the present study, univariate analysis demonstrated that intrafascicular involvement was significantly associated with postoperative complications. In contrast, a positive preoperative Tinel’s sign showed only a borderline association, while MRI-based characteristics and the use of tru-cut biopsy were not related to post-operative complication risk.

Various studies have examined the relationship between radiological and histopathological features of schwannomas; however, the diagnostic specificity of specific MRI findings—such as cystic components, degenerative changes, and perilesional edema—remains controversial [12,13,14,15]. Beyond diagnosis, these features may also have prognostic implications, potentially influencing surgical complexity and postoperative neurological outcomes. Nevertheless, studies directly evaluating the association between MRI characteristics and postoperative results are limited [5,6,7,8,16].

Ujigo and Hirai reported no significant association between homogeneous or heterogeneous signal patterns on MRI and postoperative complications [5,6]. Ahlawat et al. found perilesional edema in 87% of malignant tumors versus 18% of benign tumors, suggesting its potential discriminatory value [7]. Zhang et al. observed perilesional edema in 60% of schwannomas larger than 5 cm [8]. At the same time, Samii et al. demonstrated that perilesional edema in vestibular schwannomas may affect short-term facial nerve function without long-term impact [16]. In our series, the mean tumor volume was 9.6 ± 4.8 cm^3^. Neither perilesional edema nor degenerative changes showed a significant association with postoperative complications. Although degenerative changes tended to occur in larger schwannomas, this trend did not reach statistical significance, and perilesional edema showed no clear relationship with tumor size.

The impact of tumor volume on postoperative complications in schwannomas remains controversial [3,4,6,17,18]. Granlund et al. reported no correlation between tumor size and neurological symptoms, whereas Takahashi and Park found that larger volumes—particularly in spinal schwannomas—were associated with a higher risk of recurrence [3,17,18]. Kim et al. further suggested that large schwannomas may increase the likelihood of fascicular injury during dissection [4]. In our series, tumor volume was not significantly associated with the development of complications (*p* = 0.567). This discrepancy may be attributed to the limited sample size and heterogeneity of the present cohort. Overall, structural features of the tumor alone seem insufficient to explain complication risk, which is likely shaped by multiple interacting factors such as surgical technique and the tumor–nerve relationship.

Although tru-cut biopsy can be a useful diagnostic tool in peripheral nerve sheath tumors with suspected malignancy, it is well recognized that improper application without adherence to established algorithms may lead to serious complications [19]. Perez-Roman et al. observed new or worsened neurological deficits in 43% of patients biopsied for suspected peripheral nerve sheath tumors [20]. In contrast, Pianta et al. found a 12% incidence of transient pain exacerbation in a series of 38 cases, which they associated with smaller, superficial lesions and closer needle proximity to the nerve [21]. Similarly, Tøttrup and Graham reported no significant complications other than transient sensory loss, which resolved within two days after ultrasound-guided tru-cut biopsy [22,23]. Ozturk et al. also found no biopsy-related complications in 17 cases with indeterminate benign/malignant status and reported no correlation between the number of needle passes and pain incidence [9].

In our series, 7 of 47 patients (14.9%) underwent tru-cut biopsy, and no statistically significant association with postoperative complication risk was observed (*p* = 0.141). Considering the heterogeneity of previous reports, our results suggest that tru-cut biopsy can be safely performed when clear indications are present and the procedure is conducted in experienced centers. However, this interpretation should be cautiously made given the limited sample size.

The literature has no clear consensus on whether the fascicles constituting a schwannoma are neurologically functional [24,25]. Raj et al. reported that intrafascicular schwannomas carry an approximately 4.6-fold higher risk of postoperative sensorimotor impairment compared to extrafascicular lesions [25]. Similarly, Sawada et al. demonstrated higher rates of neurological deficits in intrafascicular resections where enucleation was not feasible [26]. In our series, the complication rate was significantly higher for intrafascicular tumors compared to extrafascicular tumors (*p* = 0.023). This finding represents a known but clinically relevant risk, underscoring the importance of carefully considering the tumor–fascicle relationship in surgical planning.

In our series, lower improvement rates were observed, with reductions at final follow-up of 82%, 64%, and 67% for pain, paresthesia, and hypoesthesia, respectively. Postoperatively, Tinel’s sign resolved in 76% of patients who had been positive preoperatively. Ujigo et al. found that patients presenting with preoperative symptoms similar to Tinel’s sign experienced more postoperative complications (*p* = 0.038) [6]. In our study, preoperative Tinel’s sign showed a near-significant association with postoperative complications (*p* = 0.084). This finding suggests that preoperative signs of nerve irritation may indicate the likelihood of symptomatic improvement and potential predictors of postoperative complication risk. However, confirmation of this association will require larger patient series and prospective studies.

Toshihide Hirai et al. reported higher complication rates in schwannomas occurring in older patients, those located in the upper extremities, and those involving major motor nerves in a cohort of 141 patients [5]. Siquera et al. observed an increased risk of complications in patients younger than 50 years and tumors larger than 3 cm [2]. In contrast, Granlund et al. reported that surgeries involving minor nerves could lead to more complications than those involving major nerves [17]. In our study, however, neither tumor volume nor anatomical location showed a significant association with the risk of complications.

This study has several limitations. The retrospective design and relatively small sample size—particularly for rare outcomes, such as postoperative complications—limited the study’s statistical power, and only univariate analyses could be performed. Although having all MRI evaluations performed by a single radiologist ensured consistency, it may have introduced observer bias. Furthermore, sensory and motor deficits were assessed clinically without quantitative measurements, which could be subject to interobserver variability. A further limitation of this study is the small size of the biopsy-only subgroup (n = 7), and findings from this group should be interpreted with caution, given the variability in the literature. These cases were inherently atypical, prompting biopsy rather than primary excision and introducing potential selection bias; therefore, the results may not be generalizable to the broader population of peripheral nerve schwannomas. These limitations should be considered when interpreting the results.

## 5. Conclusions

In this study, it is concluded that despite its benign nature, excision of a solitary schwannoma may be associated with various complications. Intrafascicular involvement was identified as the primary factor associated with postoperative complications following the intracapsular excision of benign solitary schwannomas of the extremities. In contrast, preoperative MRI characteristics, including tumor volume, perilesional edema, and degenerative changes, as well as a history of tru-cut biopsy, were not found to be clearly associated with an increased risk of postoperative complications. Radiological features alone may offer limited predictive value for postoperative neurological outcomes. Instead, the anatomical relationship between the tumor and adjacent nerve fascicles plays a more critical role in determining surgical complexity and risk. Preoperative recognition of intrafascicular tumor involvement can enhance patient selection, improve risk stratification, and support more realistic counseling regarding potential neurological outcomes.

## Figures and Tables

**Table 1:** Demographic and Radiological Characteristics of the Patients.

Characteristics	Mean ± SD
Age	38.6 ± 15
Gender	
Female	27 (57.4%)
Male	20 (42.6%)
Mean Follow-up Duration (months)	109.8 ± 65.1
Anatomic Site	
Upper Extremity	31 (65.9%)
Lower Extremity	16 (34.1%)
Mean Tumor Volume (cm^3^)	9.6 ± 4.8
Location	
Extrafascicular	34 (72.3%)
Intrafascicular	13 (27.7%)
Perilesional Edema	
Yes	15 (31.9%)
No	32 (68.1%)
Degenerative Changes	
Yes	25 (53.1%)
No	22 (46.8%)
Tru-cut Biopsy	
Yes	7 (14.9%)
No	40 (85.1%)

SD: Standard deviation.

**Table 2:** Frequency and Subtypes of Postoperative Complications.

Characteristics	Frequency (%)
Complication Status	
Yes	9 (19.1%)
No	38 (80.9%)
New-onset neurological	
Paresthesia	6 (13.2%)
Hypoesthesia	7 (14.9%)
Motor Deficit	2 (4.3%)
Other complications	
New-onset pain	4 (8.5%)
Wound Complication	1 (2.1%)
Recurrence	1 (2.1%)

**Table 3:** Comparative Analysis of Pre- and Postoperative Neurological Symptoms.

Symptom	Pre-Op (n/%)	Post-Op (n/%)	New-Onset (n/%)	Resolved (n/%)	Persistent (n/%)	Remission (n/%)
Paresthesia	37 (78.7%)	13 (27.7%)	6 (12.8%)	24 (51.1%)	7 (14.9%)	64.9
Hypoesthesia	34 (72.3%)	11 (23.4%)	7 (14.9%)	23 (48.9%)	4 (8.5%)	67.6
Pain (either local or radiating)	28 (59.6%)	5 (10.6%)	4 (8.5%)	23 (48.9%)	1 (2.1%)	82.1
Motor Deficit	3 (6.4%)	5 (10.6%)	2 (4.3%)	0 (0%)	3 (6.4%)	NA
Tinel Sign	30 (63.8%)	7 (14.9%)	0 (0%)	23 (48.9%)	7 (14.9%)	76.7

NA: Not Applicable.

**Table 4:** Univariate Analysis of Risk Factors for Postoperative Complications.

Variables	OR (95% Cl)	*p* Value (Fisher)
Degenerative Change	3.1 (0.6–14.4)	0.140
Perilesional Edema	3.5 (0.8–15.6)	0.091
Fascicular İnvolvement	5.4 (1.4–22.1)	0.037 *
Tru-Cut Biopsy	4.2 (0.81–23.9)	0.084
Preoperative Tinel Sign	3.1 (0.7–15.1)	0.141
Anatomic Site	2.55 (0.47–13.71)	0.449

* *p* < 0.05 is considered significant.

## Data Availability

The datasets generated and analyzed during the current study are available from the corresponding author on reasonable request.

## Abbreviations

The following abbreviations are used in this manuscript:

MPNTS	Malignant peripheral nerve sheath tumor
NF ½	Neurofibromatosis Type1/2
cm^3^	cubic centimeter
OR	Odds Ratio
Cl	Confidence Interval
